# Distributing questionnaires about smoking to patients: impact on general practitioners' recording of smoking advice

**DOI:** 10.1186/1472-6963-7-153

**Published:** 2007-09-24

**Authors:** Tim Coleman, Andrew Wilson, Steve Barrett, Alison Wynne, Sarah Lewis

**Affiliations:** 1 Division of Primary Care, University of Nottingham, Nottingham, UK; 2 Department of Health Sciences, University of Leicester, Leicester, UK; 3 Division of Epidemiology & Public Health, University of Nottingham, Nottingham, UK

## Abstract

**Background:**

Little is known about the impact of questionnaire-based data collection methods on the consulting behaviour of general practitioners (family physicians) who participate in research. Here data collected during a research project which involved questionnaires on smoking being distributed to patients before and after appointments with general practitioners (GPs) is analyzed to investigate the impact of this data collection method on doctors' documenting of smoking advice in medical records.

**Methods:**

Researchers distributed questionnaires on smoking behaviour to 6775 patients who attended consultations during surgery sessions with 32 GPs based in Leicestershire, UK. We obtained the medical records for patients who had attended these surgery sessions and also for a comparator group, during which no researcher had been present. We compared the documenting of advice against smoking in patient's medical records for consultations within GPs' surgery sessions where questionnaires had been distributed with those which occurred when no questionnaires had been given out.

**Results:**

We obtained records for 77.9% (5276/6775) of all adult patients who attended GPs' surgery sessions, with 51.9% (2739) being from sessions during which researchers distributed questionnaires. Discussion of smoking was recorded in 8.0% (220/2739) of medical records when questionnaires were distributed versus 4.6% (116/2537) where these were not. After controlling for relevant potential confounders including patients' age, gender, the odds ratio for recording of information in the presence of questionnaire distribution (versus none) was 1.78 (95% CI, 1.36 to 2.34).

**Conclusion:**

Distributing questionnaires about smoking to patients before and after they consult with doctors significantly increases GPs' recording of discussions about smoking medical records. This has implications for the design of some types of research into addictive behaviours and further research into how data collection methods may affect patients' and doctors' behaviours is warranted.

## Background

Questionnaires distributed to patients as they attend doctors are frequently used in primary care studies with behavioural outcomes[1]. In particular, such data collection methods have been employed in trials of smoking cessation[2,3] or health promotion interventions[4] and in some studies investigating the impact of training physicians in smoking cessation methods on doctors' clinical behaviour and patients' smoking[5]. However, little research has investigated the validity of such instruments for these uses. Post-consultation questionnaires are known to over-estimate rates of smoking cessation advice given by general practitioners (family physicians – GPs)[6,7] and hospital physicians[8,9], and false positive reports of advice increase as time elapses following patients' consultations[7,10]. There is even less research which investigates whether giving pre- or post consultation questionnaires to patients affects doctors' consulting behaviour and after extensive literature searching, the authors could identify no studies on this topic. Consequently, we analyse data from a primary care smoking cessation study, to investigate whether or not distributing questionnaires on smoking behaviour to patients before and after they see a general practitioner influenced doctors' recording of smoking cessation advice in patients' medical records.

## Method

### The Smoking Incentives Study

Data used in this analysis was collected during a study, conducted in 1997/8. This study, the 'Smoking Incentives Study (SIS)[11] investigated the impact of a new payment made to general practitioners for their health promotion activity. Data collection for SIS took place in two distinct periods which were before and after the new payment was introduced. This data collection was undertaken by research assistants (RAs) and involved merely giving out questionnaires on smoking to patients both as they waited to see their GP and after they had done so. The only other role that the RA had within the practice was to inspect medical records of patients attending surgery sessions. The RA had no interaction with the GP, but the GP could not be blinded to and was probably aware that data collection was taking place. GPs knew that questionnaires were about smoking and they could claim the new health promotion payment whenever they identified patients who had stopped smoking for three months or more. SIS was granted ethical approval by the Leicestershire Ethics Committee and had a negative result, showing that the new payment had no impact on rates of general practitioners' brief advice against smoking, as recorded on completed post-consultation questionnaires given to patients by RAs[11].

### Data collection for current analysis

This analysis is based on 32 general practitioners who participated in at least one of the data collection periods for SIS. Each GP had data collected (i.e. questionnaires distributed) at up to 12 randomly-selected surgery sessions over a 16 month period. All doctors used paper, rather than electronic, medical records, so to identify and obtain the records of patients who had attended surgery sessions, RAs used receptionists' lists which logged attending patients. For every surgery session during which data collection occurred, RAs identified comparator surgery sessions where no questionnaires had been distributed. To be selected as comparators, surgery sessions needed to be with the same general practitioner, on the same weekday and at the same time of day (i.e. morning or afternoon). Comparator surgery sessions were usually within one week of data collection ones, though this depended on whether or not a surgery with the same doctor was available at the appropriate time. Selecting a surgery with the same general practitioner was intended to eliminate inter-doctor variation in clinical behaviour and comparing surgery sessions on the same days at similar times was intended to minimise variation in numbers of patients being booked in different sessions.

RAs obtained the total number of patients attending each surgery session from receptionists' records and then sought the medical records of patients attending sessions. Where medical records could be obtained, RAs noted the age and sex of patients and also whether or not discussion of smoking was recorded on the date of the selected surgery session. RAs were trained to extract data on smoking cessation advice from medical records by a general practitioner and piloting of data extraction indicated that this was accurate. RAs inspected all available medical records of patients attending data collection surgeries and recorded whether or not there was any note that smoking had been discussed. From the brief notes inspected, it was not possible to differentiate whether doctors' advice to stop smoking had been given or merely that smoking status had been ascertained, so 'discussion of smoking' may reflect either occurrence. Inter-observer reliability of data extraction from medical records was tested using Cohen's kappa[12], comparing the data on smoking advice extracted from 100 medical records by each of two RAs with that extracted by a general practitioner.

### Analysis

We compared the medical records of patients attending surgeries where questionnaires were distributed with those where no data collection occurred to determine whether there was any difference in the proportions containing a note that smoking cessation advice had been given. We used a multi-level logistic regression model, allowing for variation at the practice, GP and clinic level, to estimate the effect of data collection procedures on recording of advice against smoking in records, with adjustment for age in quartiles, sex and whether or not the new health promotion payment had been available to general practitioners (via SIS) at the time of data collection.

## Results

We obtained and inspected medical records for 77.9% (5276/6775) of all adult patients who attended general practitioners' surgery sessions and the number of medical records inspected for each GP varied between 48 and 306. Overall, 6.4% (336/5276) of records contained a note that advice against smoking had been given. 60.2% (3178/5276) of records inspected were from women and the mean [SD] age of patients whose records were inspected was 40.7 [22.3], with age data being unavailable for 4 patients.

51.9% (2739) of medical records were from surgery sessions during which researchers distributed questionnaires on smoking behaviour and the remainder were from surgery sessions where no data collection occurred. The distribution of age and gender of patients was similar between the two groups, but the distribution of data collection surgeries differed between the 'after' period of SIS (when GPs could claim the new payment) and the 'before' period when this was not available; a researcher distributing questionnaires was present in 1271 out of 2767 (45.9%) patients seen during the 'after' period, and 1266 out of 2509 (50.5%) seen during the 'before' period. The level of agreement between both RAs and a general practitioner for the presence or absence of a note of discussion of smoking in the medical records was good[12] with the Kappa values (95% CI) for agreement between a general practitioner and the two RAs being 0.79 (0.6 to 0.98) and 0.85 (0.66 to 1.0) respectively.

Recording of advice against smoking in medical records was significantly more likely when a researcher distributed questionnaires in the surgery session; when a researcher was present 8.0% (220/2739) of medical records had a note that smoking had been discussed versus 4.6% (116/2537) when no researcher was present. (Odds ratio 1.80, 95% CI 1.38 to 2.35, p < 0.001).

Recording of advice against smoking in medical records was also more likely in female than male patients (6.9% of women's notes included a record of smoking advice versus 5.6% of men's), and in the middle two quartiles of age (9.8 and 7.5%) than in the youngest (< age 24, 3.8%) or oldest (≥ age 59, 4.3%) patients. Additionally, patients' medical records from the period of SIS before the new health promotion payment became available to general practitioners were slightly more likely to have a record of advice (6.9%) than those from the period during which this could be claimed (5.8%). This suggests that the new health promotion payment had no impact on recording of smoking advice in medical records.

Table 1 shows that, after adjustment for age, sex and for whether or not the new health promotion payment could be claimed at the time the selected consultation occurred, the odds ratio for the recording of advice against smoking in patients' medical records during surgery sessions where questionnaires were distributed compared to those where no data collection occurred was 1.78 (95% CI, 1.36 to 2.34).

**Table 1:** The effect of researcher distributing questionnaires, age, sex and availability of health promotion payment within SIS on recording of smoking advice in patient's medical records (from a multi-level logistic regression model)

	**Odds ratio (95% CI)**
**Researcher distributing questionnaires**	
**No**	1
**Yes**	1.78 (1.36 to 2.34)
	
**Males**	1
**Female**	1.16 (0.91 to 1.48)
	
**Age (in quartiles)**	
**0 to 24**	1
**24 to 40**	2.79 (1.98 to 3.94)
**40 to 59**	2.12 (1.48 to 3.04)
**59 to 93**	1.19 (0.80 to 1.78)
	
**Payment available in SIS study**	
**No**	1
**Yes**	0.90 (0.68 to 1.18)

## Discussion

This analysis demonstrates that when researchers distribute questionnaires about smoking to patients before and after they see their general practitioner and the doctor is not blind to questionnaire contents, he or she is almost twice as likely to record discussion of smoking in the medical records. The impact of the researcher had an independent effect which was not affected by patients' age or sex or the availability to general practitioners of a new smoking-related health promotion payment which was being evaluated[13].

The authors searched but could find no papers investigating the impact on general practitioners' behaviour of questionnaires distributed to patients, so we believe that this study is original. Additionally, data presented here was collected at a time when patients in the UK were not required to give consent for medical researchers to inspect their medical records for research purposes (i.e. to 'opt in' to this type of research). If the study were repeated today, researchers would need to contact patients and obtain their consent before inspecting records, introducing potential bias into this process. A weakness of the study is that researchers were only able to obtain 78% of the medical records for patients who attended selected surgery sessions and we can say nothing about the records which were not obtained. Researchers relied on clerical staff to find records in addition to performing their usual daily work tasks and it is possible that this unpaid work did not receive high priority, resulting in the relatively high rate of non-located records. It is unlikely, however, that researchers' inability to locate medical notes would be related to doctors' recording of discussing smoking, so data from records which researchers could not access would not be expected to affect study findings. Finally, it is possible that unmeasured factors may have affected doctors' documentation of the smoking advice that they delivered to patients. For example, doctors are more likely to advise smokers' who are interested in stopping or who present with smoking-related problems[14]. It is possible, therefore, that doctors' propensity to discuss smoking when it may be relevant to patients' presenting problems, or with 'interested' smokers, results in more discussion of smoking between GPs and smokers than non-smoking patients. If we had collected data on patients' smoking status in both study groups, then we would have been able to adjust our findings for any influence that this may have had. However, our method for selecting comparison group surgeries should have ensured that these were similar and seems unlikely that smoking prevalence would differ significantly between patients attending the two groups of surgeries, such that this could explain study findings.

General practitioners do not document all of the advice against smoking which they deliver to patients[6], so questionnaires may have prompted more discussion of smoking by GPs, more recording of advice or both of these outcomes. One could differentiate between these possibilities by either directly observing or recording what actually occurred in consultations, but recording of consultations can also influence consulting behaviour[15]. Researchers who plan to use pre- or post-consultation questionnaires need to consider the potential impact of these on doctors' behaviours. It is likely that questionnaires addressing other topics (e.g. alcohol or dietary intake) may have similar impacts on other aspects of clinical behaviour (e.g. discussions about alcohol or recording of these). Such impacts are likely to be most important in studies where recording of doctors' behaviours in medical records are used as outcomes. In these kinds of study, questionnaires use could result in higher overall rates of outcomes than anticipated, affecting sample sizes need to show differences between groups.

Researchers also need to be cautious when using pre-consultation questionnaires in primary care trials that involve allocating participants to "normal care" treatment groups. Some primary care smoking cessation studies have used "normal care" treatment arms[2,16-18], but questionnaires distributed to general practitioners in 'normal care' trial arms would be expected to increase doctors' recording of smoking status and may even influence doctors' delivery of smoking advice to patients. As doctors' brief advice against smoking is an effective smoking cessation intervention[19], such questionnaires could potentially increase cessation rates in 'normal care' trial arms, interfering with the power of any trial to detect differences between trial groups.

This study provides direct evidence for the value of blinding study participants, including health professionals, to the contents of research questionnaires[20]. However, in applied health services research this is not always possible because doctors will often wish to see questionnaires to be reassured that data collection instruments are not too long and will not cause offence to patients[21]. Further research into the impact of questionnaires distributed to patients for research purposes is required and where blinding of research participants is not possible, researchers must consider the likely implications of this.

## Conclusion

When researchers distribute questionnaires to patients before and after they consult with GPs and GPs are aware of questionnaire content, this can affect GPs' behaviour. Giving out questionnaires which asked about patients' smoking habits resulted in GPs recording more advice against smoking in patients' medical records. This increase could have been due to an increase either in the actual amount of smoking cessation advice delivered or in the recording of advice given. Study findings require consideration when designing research studies that use data recorded in medical records as outcome measures.

## Competing interests

The author(s) declare that they have no competing interests.

## Authors' contributions

TC, SL and AW designed the analysis and TC had the idea for the analysis. AW and SB collected the data for the study and SL performed the statistical analysis. All authors were involved in writing the paper and interpreting results. TC is the guarantor. All authors read and approved the final manuscript.

## Pre-publication history

The pre-publication history for this paper can be accessed here:


